# Long COVID and Acute Stroke in the Emergency Department: An Analysis of Presentation, Reperfusion Treatment, and Early Outcomes

**DOI:** 10.3390/jcm14186514

**Published:** 2025-09-16

**Authors:** Daian-Ionel Popa, Florina Buleu, Aida Iancu, Anca Tudor, Carmen Gabriela Williams, Marius Militaru, Codrina Mihaela Levai, Tiberiu Buleu, Livia Ciolac, Anda Gabriela Militaru, Ovidiu Alexandru Mederle

**Affiliations:** 1 Research Center for Medical Communication, Victor Babes University of Medicine and Pharmacy, 300041 Timisoara, Romania; daian-ionel.popa@umft.ro (D.-I.P.); codrinalevai@umft.ro (C.M.L.); 2 Doctoral School, Faculty of General Medicine, Victor Babes University of Medicine and Pharmacy, 300041 Timisoara, Romania; williams.carmen@umft.ro (C.G.W.); livia.ciolac@umft.ro (L.C.); 3 Emergency Municipal Clinical Hospital, 300254 Timisoara, Romania; marius.militaru@umft.ro (M.M.); militaru.anda@umft.ro (A.G.M.); mederle.ovidiu@umft.ro (O.A.M.); 4 Department of Cardiology, Victor Babes University of Medicine and Pharmacy, E. Murgu Square No. 2, 300041 Timisoara, Romania; 5 Department of Radiology, Victor Babes University of Medicine and Pharmacy, E. Murgu Square No. 2, 300041 Timisoara, Romania; aida.parvu@umft.ro; 6 Department of Functional Sciences, Victor Babes University of Medicine and Pharmacy, E. Murgu Square No. 2, 300041 Timisoara, Romania; atudor@umft.ro; 7 Department of Neuroscience, Discipline of Neurology II, Victor Babes University of Medicine and Pharmacy, E. Murgu Square No. 2, 300041 Timisoara, Romania; 8 Faculty of Nursing, Victor Babes University of Medicine and Pharmacy, 300041 Timisoara, Romania; tiberiu.buleu@umft.ro; 9 Department of Internal Medicine I, Medical Semiology I, Victor Babes University of Medicine and Pharmacy Timisoara, Eftimie Murgu Square No. 2, 300041 Timisoara, Romania; 10 Center of Advanced Research in Cardiology and Hemostasology, University of Medicine and Pharmacy Victor Babes Timisoara, E. Murgu Square, Nr. 2, 300041 Timisoara, Romania; 11 Department of Surgery, Emergency Discipline, Victor Babes University of Medicine and Pharmacy, 300041 Timisoara, Romania

**Keywords:** Long COVID, code stroke alert, emergency department, ED time targets, outcome

## Abstract

**Background and Objectives:** Long COVID has been linked with persistent neurological symptoms, but data on its effects on acute stroke presentation, management, and outcomes remain unclear. This study aimed to compare the clinical profile, management, and short-term outcome of acute ischemic stroke patients with and without Long COVID. **Materials and Methods:** A retrospective cohort study was conducted on 132 patients who presented at admission with code stroke alert in our Emergency Department (ED). Out of those, 26 were identified to have the Long COVID condition and assigned to the Long COVID group, and 106 were without the Long COVID condition and assigned to the No Long COVID group. Baseline demographics, stroke severity by NIHSS (National Institutes of Health Stroke Scale), risk factors, admission symptoms, laboratory findings, Emergency department time targets, reperfusion treatments received, and outcomes between the two groups were compared. **Results:** There were no significant differences between the two groups in age, gender, baseline NIHSS scores, ED time targets, or laboratory values. The proportion of patients with Long COVID significantly increased among non-smokers (Fisher’s Exact Test chi-squared, *p* = 0.027). Also, patients suffering from Long COVID exhibited higher incidences of headache (19.2% compared to 5.7%, OR = 3.97, *p* = 0.040) and facial drooping (42.3% compared to 19.8%, OR = 2.97, *p* = 0.022). The mechanical thrombectomy was more frequent among the group with Long COVID (30.8% vs. 16.0%), but this difference was not statistically significant. More hemorrhagic transformations happened in the Long COVID group (26.9% vs. 14.2%, *p* = 0.143). Discharge rates and hospital length of stay in days were similar between groups. **Conclusions:** Long COVID patients did not present notable differences in emergency department time targets, baseline stroke severity, or short-term outcomes when presenting with code stroke alert. Nevertheless, specific clinical characteristics—such as elevated occurrences of headache and facial drooping—were more frequently observed in patients with Long COVID, alongside non-significant trends indicating a greater utilization of mechanical thrombectomy and increased rates of hemorrhagic transformation. These results imply that Long COVID may have a subtle impact on stroke presentation and potentially on underlying cerebrovascular susceptibility. Further prospective studies with larger sample sizes are necessary to investigate Long COVID’s long-term neurological and vascular consequences.

## 1. Introduction

Since the COVID-19 pandemic began, evidence has grown regarding the multisystem, long-term effects of SARS-CoV-2 infection—conditions now popularly known as Long COVID or post-acute sequelae of COVID-19 (PASC) [1]. The typical symptoms include fatigue and shortness of breath. Still, they can also include cognitive dysfunction, chest pain, and a variety of multi-system complaints that persist for weeks or months, well beyond the acute phase of infection [2,3]. Although initially considered primarily a respiratory illness [4], COVID-19 has demonstrated substantial neurovascular implications, including an increased risk of ischemic and hemorrhagic stroke [5], even in patients recovered from COVID-19 infection [6].

Stroke is one of the leading causes of mortality and long-term disability worldwide [7]. Effective management of acute stroke relies heavily on prompt diagnosis, imaging, and access to specialized care, which is usually provided in a dedicated stroke unit or through neurological support. Nevertheless, in settings with limited resources or general hospitals lacking neurologists or stroke teams, the responsibility for stroke care often rests with emergency department (ED) physicians—frequently without the benefit of subspecialty consultation [8,9,10]. The presence of Long COVID symptoms in patients presenting with stroke adds a layer of complexity, potentially affecting both clinical presentation and patient outcomes [11].

The new findings suggest that Long COVID may alter cardiovascular and coagulation profiles, precipitate endothelial dysfunction, and increase systemic inflammation—all factors relevant to stroke [12]. However, Long COVID’s clinical and logistical impact in the acute stroke setting remains poorly characterized, particularly in facilities without advanced stroke infrastructure. Understanding how Long COVID interacts with acute stroke in such environments is essential for improving triage, treatment, and outcomes.

This study aims to assess the clinical characteristics, management parameters, and outcomes of patients presenting with acute stroke in an emergency department lacking neurologists or a dedicated stroke unit, with a specific focus on those with a history of long COVID. By comparing patients with and without Long COVID, we seek to identify potential differences in presentation, ED time targets, and short-term outcomes, and highlight the unique challenges that Long COVID poses in real-world emergency stroke care.

## 2. Materials and Methods

### 2.1. Study Design

This observational study employed a retrospective cohort design. It included patients with acute stroke alert who presented to the ED of a general hospital in Timișoara, Romania, between November 2020 and May 2023. Timișoara is a city with approximately 300,000 residents. The hospital is the second-largest healthcare facility in the county, managing over 30,000 ED visits annually. Notably, it operates without on-call neurologists or a dedicated stroke unit.

### 2.2. Definition of Long COVID

According to the World Health Organization (WHO) [3] criteria, Long COVID was defined as the persistence of new, returning, or ongoing symptoms for more than four weeks after the initial SARS-CoV-2 infection, not attributable to other causes. Common symptoms considered included fatigue, dyspnea, cognitive impairment (“brain fog”), chest pain, and palpitations. This diagnosis was made either by preexisting medical documentation or retrospective clinical interviews elicited during hospitalization or thereafter in follow-up. Only patients with adequate documentation to establish or negate the diagnosis of Long COVID were considered.

### 2.3. Study Population

A total of 197 patients eligible for cerebral reperfusion (only patients with acute ischemic stroke) were admitted to our ED with code stroke alert during this period. From them, we included in the final sample patients ≥ 18 years old, who had complete clinical, laboratory, and imaging data, and sufficient documentation to determine Long COVID status from medical records or retrospective interviews.

**Figure 1:**
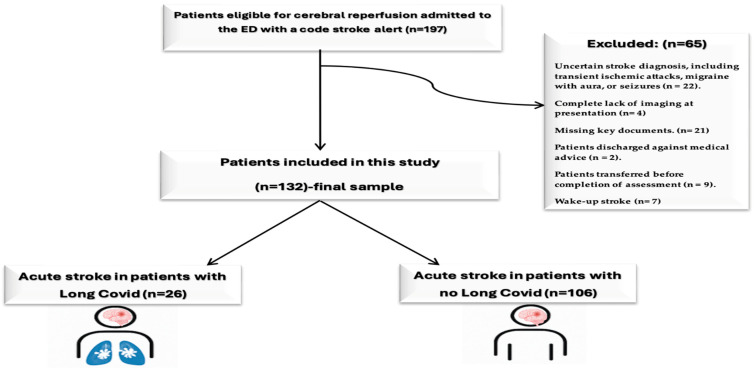
Study flowchart (created in BioRender; Popa, D., 2025; https://BioRender.com/8dpdhqz, accessed on 2 September 2025).

Patients were excluded if they had an uncertain stroke diagnosis, including transient ischemic attacks, migraine with aura, or seizures (*n* = 22); complete lack of imaging at presentation (*n* = 4); incomplete or non-analyzable records, such as missing key documents (*n* = 21); were discharged against medical advice (*n* = 2); were transferred before completion of assessment (*n* = 9); had a wake-up stroke (*n* = 7); or were under 18 years of age. After applying inclusion and exclusion criteria, a final cohort consisting of 132 patients with acute ischemic stroke was formed as the final sample. These were divided into two groups: patients with acute stroke and Long COVID (*n* = 26) and patients with acute stroke and No Long COVID (*n* = 106) (Figure 1).

Data were retrospectively extracted from electronic medical records and included the following variables: demographics (age, gender, and smoking history); clinical parameters, including National Institutes of Health Stroke Scale (NIHSS) scores at admission and 24 h post-presentation; ischemic stroke etiology classified according to the TOAST (Trial of Org 10172 in Acute Stroke Treatment) criteria; and presenting symptoms such as aphasia, hemiparesis, and headache. According to our national protocol, NIHSS scores were assessed at admission and again at 24 h post-admission to evaluate early neurological changes, including early improvement or deterioration, which are significant predictors of short-term outcomes in acute stroke patients.

Time metrics (measured in minutes) were also recorded, including onset-to-door time, door-to-physician time, door-to-CT time, and door-in–door-out time. Upon the patient’s arrival at the ED, documentation was made regarding the time of onset of stroke symptoms as reported by the patient/their relatives, or caregiver. In instances where symptoms began during sleep, the last known time without symptoms was recorded, while cases in which patients awoke with new symptoms were classified as “wake-up stroke.” The duration from symptom onset to ED arrival was referred to as onset-to-ED door, and the term door-in-door-out time was used to indicate the departure from the ED for transfer of the patient to a hospital with an Acute Stroke Unit. The target for door-in-door-out time was established at ≤120 min, aligning with the recommendations of The Joint Commission and the Brain Attack Coalition for transferring patients to hospitals equipped with a stroke team [13]. This guideline is also adhered to in our national protocol [14]. Additionally, other time targets set forth by our national protocol analyzed in our study included door-to-physician time within ≤10 min and door-to-CT time within ≤25 min.

Comorbidities were documented (from the patient’s anamnesis and medical history) and included hypertension, diabetes mellitus, atrial fibrillation, dyslipidemia, prior stroke, and alcohol use. Laboratory data comprised hemoglobin levels, platelet count, international normalized ratio (INR), activated partial thromboplastin time (APTT), and blood glucose. Treatment modalities included intravenous thrombolysis and endovascular thrombectomy. Furthermore, smoke and alcohol intake were extracted retrospectively from the electronic medical records, with both variables coded as binary (Yes/No). A “Yes” value for smoking indicated that the patient had any documented history of tobacco use (current or past), while “No” stated no documented history. Similarly, “Yes” for alcohol intake referred to any documented alcohol consumption, without further specification of frequency, duration, or quantity. Outcomes assessed were hemorrhagic transformation, discharge status, and total length of hospitalization. The total length of hospitalization was measured in days and calculated from admission to the Neurology Department until discharge or death.

### 2.4. Radiological Assessment

All patients underwent urgent neuroimaging upon ED arrival according to the national “stroke code alert” protocol. The primary imaging modality was non-contrast brain CT, performed using a 128-slice Siemens Somatom Xcite syngo CT VA40 (Siemens Healthineers, Erlangen, Germany). This helped to rule out intracranial hemorrhage, and depict early ischemic changes—sulcal effacement, loss of gray-white differentiation—as well as mass lesions and structural abnormality.

In selected cases with suspected large vessel occlusion (LVO), CT angiography (CTA) of the head and neck was performed to assess for arterial occlusion, stenosis, or dissection. Magnetic resonance imaging (MRI) was not used in the acute diagnostic phase due to its inaccessibility in our hospital’s emergency setting. All imaging studies were interpreted by on-call radiologists and confirmed stroke cases were referred to a neurology and stroke team at a secondary center for further evaluation and management.

### 2.5. Statistical Analysis

Statistical analysis was conducted using JASP version 0.19.3 (University of Amsterdam). Continuous variables were tested for normality using the Shapiro–Wilk test. Depending on the distribution, data were presented as mean ± standard deviation (SD) for normally distributed variables or as median with interquartile range (Q1–Q3) for non-normally distributed variables. Comparisons between groups were performed as follows: for non-normally distributed numerical variables, the Mann–Whitney U test was applied, and for categorical variables, either the Chi-square test or Fisher’s exact test was used, depending on cell counts, following the methodological framework described by Kim HY [15]. A survival analysis evaluated hospitalization duration and discharge outcomes in patients with and without Long COVID. The independent samples *t*-test was applied for group comparisons involving normally distributed numerical variables. A *p*-value < 0.05 was considered to indicate statistical significance. Logistic regression was used to calculate odds ratios (OR) with 95% confidence intervals (CI) for categorical variables that showed significant differences between groups. This method allowed us to quantify the strength of association between Long COVID and specific symptoms (e.g., headache, facial drooping, smoking status).

## 3. Results

### Demographic Characteristics

A total of 132 stroke patients were included in the analysis, of whom 26 (19.7%) had Long COVID and 106 (80.3%) did not. There were no statistically significant differences between the Long COVID and No Long COVID groups in terms of age (68.27 ± 12.82 vs. 67.83 ± 13.19 years, *p* = 0.922), systolic blood pressure (147.89 ± 17.46 vs. 152.35 ± 21.27 mmHg, *p* = 0.311), or diastolic blood pressure (79.23 ± 15.34 vs. 80.17 ± 13.68 mmHg, *p* = 0.944) at admission. Gender distribution was not significantly different between groups, with females comprising 57.7% of the Long COVID group and 43.4% of the No Long COVID group (*p* = 0.272). The mode of arrival (EMS (Emergency Medical Services) vs. private car) and time of presentation (day vs. night shift) were also similar between groups (*p* = 0.770 and *p* = 1.000, respectively). As measured by the NIHSS at admission and 24 h post-admission, stroke severity showed no statistically significant differences between groups. Although NIHSS scores were numerically higher in the Long COVID group at each time point, the differences were not statistically significant (e.g., NIHSS at admission: 13.62 ± 4.3 vs. 12.11 ± 5.24, *p* = 0.162). The distribution of baseline NIHSS severity categories did not differ significantly (*p* = 0.215). There were no statistically significant differences between groups regarding stroke etiologies by TOAST (Trial of Org 10172 in Acute Stroke Treatment) classification (*p*=0.400). In both groups, cardioembolism was the leading subtype (34.6% vs. 33.0%). Larger-artery atherosclerosis existed more frequently in the Long COVID group, and small-vessel occlusion occurred less regularly here; none reached significance (19.2% vs. 7.5%, 7.7% vs. 16%, respectively). (Table 1).

Comparison of medical history and risk factors revealed no significant differences between stroke patients with Long COVID (*n* = 26) and those without (*n* = 106) for most variables. The prevalence of hypertension was nearly identical in both groups (69.2% vs. 69.8%, *p* = 1.000), as were rates of diabetes mellitus (46.2% vs. 36.8%, *p* = 0.380), atrial fibrillation (23.1% vs. 13.2%, *p* = 0.581), dyslipidemia (42.3% vs. 35.8%, *p* = 0.651), and previous stroke history (7.7% vs. 8.5%, *p* = 1.000). Similarly, alcohol consumption was comparable between groups (23.1% vs. 18.9%, *p* = 0.593). However, smoking was significantly less common among Long COVID patients (3.8%) compared to those without Long COVID (22.6%) (*p* = 0.027), suggesting a potential inverse association between smoking and Long COVID in this stroke population. Caution is warranted in interpretation due to small subgroup sizes. Regarding neurological symptoms at presentation, most features were comparable across groups. Other symptoms—including hemiparesis, aphasia, dysarthria, sudden vision problems, and loss of consciousness—did not differ significantly between groups (all *p* > 0.19) (Table 2).

The proportion of patients with Long COVID was significantly higher among non-smokers (Fisher’s Exact Test, *p* = 0.027), indicating that smoking appeared to be a protective factor (OR = 0.14, 95% CI [0.02, 0.96]). Among patients with Long COVID, the occurrence of headaches was significantly increased (*p* = 0.040), with Long COVID being a risk factor for headache (OR = 3.97, 95% CI [1.11, 14.23]). Similarly, the prevalence of facial drooping was significantly higher in patients with Long COVID (*p* = 0.022), where Long COVID was identified as a risk factor (OR = 2.97, 95% CI [1.19, 7.40]) (Table 2).

Time interval comparisons between stroke patient groups revealed no statistically significant differences across all measured in-hospital time metrics. The onset-to-ED-door time was similar in both groups (188.85 ± 45.02 vs. 194.39 ± 53.67 min, *p* = 0.696), indicating comparable delays in seeking care after stroke symptom onset.

Likewise, the door-to-physician time (5.81 ± 2.93 vs. 5.29 ± 2.98 min, *p* = 0.465) and door-to-CT scan time (21.39 ± 19.67 vs. 22.38 ± 18.96 min, *p* = 0.624) did not differ significantly between the groups. Finally, the door-in-door-out time, which reflects the efficiency of patient transfer processes, was nearly identical in the two groups (50.42 ± 18.55 vs. 49.41 ± 18.34 min, *p* = 0.639) (Table 3).

No significant differences in routine hematologic, metabolic, or coagulation lab values between those with and those without Long COVID were noted. Laboratory profiles at admission are to be considered as similar (Table 4).

There was no statistically significant difference between reperfusion treatments. Most patients from both groups did not receive acute reperfusion therapy, 61.5% from the Long COVID group and 70.8% from the No Long COVID group. Small proportions from both groups received intravenous thrombolysis, 7.7% versus 13.2%. Mechanical thrombectomy showed a higher frequency in the Long COVID group, 30.8%, compared to 16.0% in the No Long COVID group, though not statistically significant. The clinical outcomes of these two groups were also similar. Most patients were finally discharged, with a discharge rate of 92.3% for Long COVID against 90.1% for No Long COVID (*p* = 1.000). The mean length of hospitalization expressed as mean ± SD days was almost identical (8.27 ± 2.24 vs. 8.38 ± 2.73, *p* = 0.956). A higher percentage of hemorrhagic transformation was found in the Long COVID group compared to the other group, i.e., 26.9% against 14.2% but it was not statistically significant (*p* = 0.143) (Table 5).

**Figure 2:**
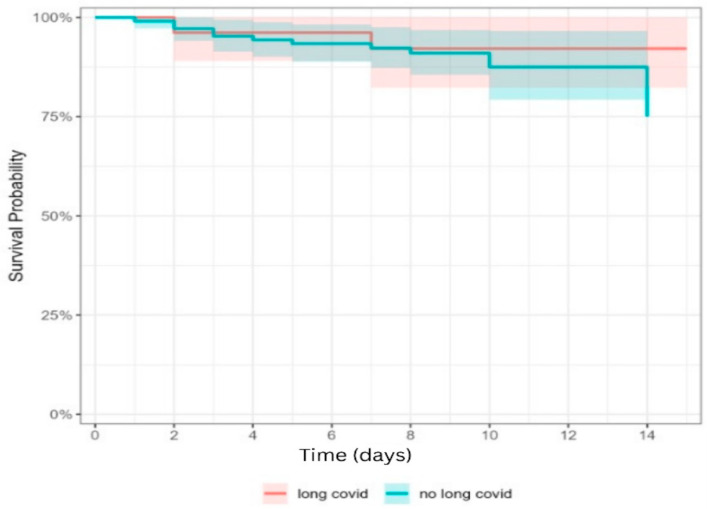
Kaplan–Meier survival plot comparing the two groups (*n* = 132).

Survival rates remain high in both groups over the 14-day follow-up period, exceeding 75%. The survival curves are closely aligned, indicating no substantial difference in short-term survival between stroke patients with Long COVID-19 and those without. The shaded areas, representing 95% confidence intervals, overlap almost entirely, suggesting that any observed differences are not statistically significant. A slight dip in survival is observed after day 10 in the group without Long COVID, but this remains well within the confidence interval of the Long COVID group (Figure 2).

## 4. Discussion

In this retrospective cohort study, we examined the clinical characteristics, management strategies, and short-term outcomes of patients experiencing acute ischemic stroke, both with and without Long COVID. Our findings suggest that while overall stroke management care metrics and outcomes did not significantly differ between the groups, patients with Long COVID exhibited distinct symptom patterns and trends in intervention and complications, potentially reflecting subtle post-COVID-19 neurological effects.

A significant finding was the notably lower prevalence of smoking history among individuals diagnosed with Long COVID. This disparity may indicate differences in foundational health behaviors between those susceptible to Long COVID and those not. Alternatively, it could be attributed to lifestyle changes enacted after COVID-19 infection, such as smoking cessation driven by heightened health consciousness or the respiratory issues encountered during the acute phase [16]. Previous research has indicated that individuals often reduce or eliminate smoking after experiencing severe illnesses, particularly respiratory infections, due to apprehensions regarding long-term health consequences and the aggravation of symptoms [17]. Furthermore, smokers may be underrepresented in the Long COVID cohort, as they may have been more prone to experiencing severe acute outcomes from COVID-19, including increased mortality [18].

A significantly greater proportion of patients experiencing headaches was noted among those with Long COVID compared to those without (Fisher’s Exact Test, *p* = 0.040). Through logistic regression analysis, Long COVID was determined to be a significant risk factor for headache, presenting an odds ratio (OR) of 3.97 (95% CI [1.11, 14.23]), which suggests that individuals with Long COVID are nearly four times more likely to report headaches. This observation is consistent with the existing literature that recognizes headache as one of the most common and enduring neurological symptoms among individuals suffering from post-acute sequelae of SARS-CoV-2 infection [19,20]. It has been hypothesized that headaches associated with Long COVID may arise from persistent low-grade neuroinflammation, dysregulation of the trigeminovascular system, or autonomic dysfunction [21,22]. Such mechanisms could lead to heightened central sensitization or changes in cerebral perfusion, contributing to headache after an acute cerebrovascular event. Consequently, the elevated incidence of headache within our cohort may indicate an underlying post-viral pathophysiology rather than variations in stroke subtype or severity.

Another finding indicated that the percentage of patients exhibiting facial drooping was markedly higher among those diagnosed with Long COVID than those without this condition (Fisher’s Exact Test, *p* = 0.022). The logistic regression analysis indicated that Long COVID serves as a notable risk factor for facial drooping, presenting an odds ratio (OR) of 2.97 (95% CI [1.19, 7.40]), which implies that individuals suffering from Long COVID were nearly three times more likely to display this symptom at the onset of a stroke. Although facial drooping is a well-recognized indicator of anterior circulation stroke, particularly involving the middle cerebral artery, its heightened occurrence in Long COVID patients may signify a transformation in symptom manifestation, potentially influenced by post-viral neuroinflammatory mechanisms or vascular endothelial dysfunction [23,24]. Such alterations could render patients more susceptible to significant cranial nerve involvement or changes in the presentation of ischemic symptoms; however, additional imaging-based research is required to validate this correlation.

Although no statistically significant differences were observed in metrics related to the ED time targets, reperfusion treatment rates, or most laboratory parameters, a greater percentage of patients within the Long COVID cohort received mechanical thrombectomy. While not statistically significant, this observation prompts an inquiry into whether Long COVID could predispose individuals to more proximal or large-vessel occlusions. This potential predisposition may be attributable to ongoing endothelial dysfunction or hypercoagulability—mechanisms associated with both acute COVID-19 and its post-acute sequelae [25,26].

Moreover, a greater incidence of hemorrhagic transformation was noted in the Long COVID cohort compared to the control group, although this variation did not achieve statistical significance. Nonetheless, the observed trend may possess clinical importance, particularly in light of emerging evidence linking Long COVID to ongoing endothelial dysfunction, heightened platelet activation, and disrupted coagulation pathways [27]. These pathophysiological alterations could heighten the risk of complications related to reperfusion, such as hemorrhagic transformation, especially after thrombolytic or endovascular interventions. Although the current study’s sample size limits the power to detect a definitive association, the observed difference supports the hypothesis that post-COVID vascular abnormalities could influence outcomes in acute stroke care. Larger, prospective studies are warranted to investigate the interaction between Long COVID-related vascular changes and cerebrovascular reperfusion strategies.

Considering these trends, no statistically significant differences were found in discharge outcomes or hospital length of stay for patients with Long COVID compared to those without, indicating that short-term recovery trajectories were broadly similar. This observation is consistent with the findings of Al-Aly et al. [28], who noted that while individuals with a history of COVID-19 infection exhibited an elevated long-term risk for cerebrovascular and cardiovascular events, these risks did not necessarily correspond with poorer immediate outcomes in patients who suffered an acute stroke. The differentiation between acute and chronic effects emphasizes the intricacies of post-COVID vascular pathology. It underscores the importance of distinguishing between short-term functional recovery and long-term vascular risk.

Our findings are consistent with emerging evidence from recent studies. For instance, Qureshi et al. [29] identified an elevated risk of stroke following COVID-19 infection, particularly during the initial months of recovery. Nonetheless, their study, like several others, concentrated mainly on the incidence of stroke. In contrast, our research contributes to the existing literature by exploring the clinical characteristics, management, and short-term outcomes of patients who have previously suffered a stroke within the framework of Long COVID. Comparably, a retrospective cohort investigation conducted by Siegler et al. [30] indicated increased mortality and disability among stroke patients with a history of COVID-19. It is crucial to acknowledge, however, that their cohort comprised patients with active infections and more severe systemic illnesses. These factors may have influenced poorer outcomes and consequently limited direct comparisons with our Long COVID population, beyond the acute phase of infection.

## 5. Study Strengths and Limitations

This study has several strengths. It is one of the initial investigations to specifically explore the clinical profile, emergency management, and short-term outcomes associated with acute ischemic stroke in patients suffering from Long COVID, thereby providing valuable insights that extend beyond the current literature, which predominantly emphasizes stroke incidence. The application of stringent inclusion criteria—mandating comprehensive clinical, imaging, and laboratory data—ensured the reliability of the data and reduced the occurrence of missing information. The utilization of standardized instruments, such as the NIHSS, along with the analysis of emergency department time targets for acute stroke management (for instance, door-to-CT time), enhanced the consistency and comparability of the results. Furthermore, the study considered significant confounding variables, including age, sex, and vascular risk factors, allowing for a more concentrated evaluation of the potential impact of Long COVID on stroke characteristics. The identification of unique clinical features, such as elevated rates of headache and facial drooping within the Long COVID cohort, offers novel insights that may inform future diagnostic and therapeutic strategies for post-COVID stroke populations.

This study also has limitations. Its retrospective design carries risks of selection bias, incomplete data, and limited control over confounders, particularly for Long COVID diagnosis. The small sample size, especially within the Long COVID subgroup, may have reduced statistical power, preventing some trends—such as hemorrhagic transformation or thrombectomy rates—from reaching significance. As a single-center study, generalizability is limited, given potential differences in patient demographics, stroke protocols, and post-COVID care. Only short-term, in-hospital outcomes were assessed, without evaluation of long-term neurological recovery, functional independence, or recurrent stroke risk. Post-acute anticoagulation therapy, which could affect stroke presentation or complications, was not systematically recorded. Smoking and alcohol use were captured from ED records as binary variables, limiting precision. Key modifiers of Long COVID—time since infection, severity of the acute illness, and vaccination status—were inconsistently documented. Variability in Long COVID definitions and symptom reporting may have further affected case classification.

Despite these limitations, the study provides valuable insights into the short-term cerebrovascular profile of patients with and without Long COVID. Future prospective research using standardized definitions, biomarkers, and comprehensive follow-up is needed to clarify outcomes and risk factors.

## 6. Conclusions

This study offers initial evidence indicating that Long COVID might affect both the clinical presentation and progression of acute ischemic stroke, while not significantly impacting essential emergency care metrics or short-term outcomes. Discharge rates and the duration of hospitalization were comparable across the groups; however, patients with Long COVID exhibited symptoms such as headache and facial drooping more frequently and showed non-significant trends toward elevated rates of mechanical thrombectomy and hemorrhagic transformation. These results imply that, although Long COVID does not seem to interfere with acute stroke pathways regarding emergency department time targets or immediate prognosis, it may be linked to subtle neurological and vascular changes that influence stroke phenotypes. Identifying these symptom patterns could be particularly crucial in settings with limited resources, where detailed clinical evaluations inform decision-making. Due to the retrospective nature of this study and its constrained sample size, future prospective multicenter research with standardized diagnostic criteria and long-term follow-up is necessary to fully assess the cerebrovascular effects of Long COVID and guide triage and treatment strategies for this emerging patient subgroup.

## Figures and Tables

**Table 1:** Baseline characteristics of study patients (*n* = 132).

Variables		Sample		*p* Value
		Long COVID (*n* = 26)	No Long COVID (*n* = 106)	
**Demographics and Baseline Stroke Severity**				
Age, years		68.27 ± 12.82 69 (64–75.75)	67.83 ± 13.19 70 (62–78)	0.922
SBP, mmHg		147.89 ± 17.46 150 (135.5–159)	152.35 ± 21.27 150 (140–170)	0.311
DBP, mmHg		79.23 ± 15.34 80 (72.5–84.75)	80.17 ± 13.68 80 (70–90)	0.944
Gender	Male	11 (42.3%)	60 (56.6%)	0.272
	Female	15 (57.7%)	46 (43.4%)	
Shift	Day	20 (76.9%)	80 (75.5%)	1.000
	Night	6 (23.1%)	26 (24.5%)	
Arrival mode	Emergency Medical Services	21 (80.8%)	89 (83.9%)	0.770
	Private car	5 (19.2%)	5 (21.7%)	
NIHSS on admission		13.62 ± 4.3 13 (11–15.75)	12.11 ± 5.24 11 (8–16)	0.162
NIHSS at 24H		9.58 ± 5.46 8.5 (6.25–12.75)	10.86 ± 7.45 10 (4–17)	0.569
NIHSS Severity at admission to the ED	Severe 21–42	3 (11.5%)	6 (5.7%)	0.215
	Moderate to severe 16–20	3 (11.5%)	24 (22.6%)	
	Moderate 5–15	20 (76.9%)	69 (65.1%)	
	Mild 1–4	0 (0.0%)	7 (6.6%)	
TOAST Etiology	Stroke of Undetermined	8 (30.8%)	37 (34.9%)	0.400
	Stroke of Other Determined	2 (7.7%)	9 (8.5%)	
	Small-Vessel Occlusion	2 (7.7%)	17 (16.0%)	
	Large-Artery Atherosclerosis	5 (19.2%)	8 (7.5%)	
	Cardioembolism	9 (34.6%)	35 (33.0%)	

For numerical: mean ± SD; median (Q1–Q3); for nominal: *n* (%).

**Table 2:** Medical history and risk factors for all patients (*n* = 132).

Variables	Sample		*p* Value
	Long COVID (*n* = 26)	No Long COVID (*n* = 106)	
**Medical History and Risk factors**			
Hypertension	18 (69.2%)	74 (69.8%)	1.000
Diabetes mellitus	12 (46.2%)	39 (36.8%)	0.380
Atrial Fibrillation	6 (23.1%)	14 (13.2%)	0.581
Dyslipidemia	11 (42.3%)	38 (35.8%)	0.651
Previously stroke	2 (7.7%)	9 (8.5%)	1.000
Current smoking	1 (3.8%)	24 (22.6%)	0.027 *
Current alcohol consumption	6 (23.1%)	20 (18.9%)	0.593
**Neurological symptoms on admission**			
Hemiparesis	9 (34.6%)	31 (29.2%)	0.637
Aphasia	3 (11.5%)	26 (24.5%)	0.192
Dysarthria	5 (19.2%)	18 (17.0%)	0.777
Headache	5 (19.2%)	6 (5.7%)	0.040 *
Facial drooping	11 (42.3%)	21 (19.8%)	0.022 *
Sudden vision problems	1 (3.8%)	12 (11.3%)	0.462
Loss of consciousness	1 (3.8%)	9 (8.5%)	0.686

For nominal: *n* (%), * Significant difference.

**Table 3:** Acute stroke management time targets in the Emergency Department (*n* = 132).

Variables	Sample		*p* Value
	Long COVID (*n* = 26)	No Long COVID (*n* = 106)	
Emergency Department time targets			
Onset-to-ED-door time (minutes)	188.85 ± 45.02 190 (157.5–220)	194.39 ± 53.67 200 (151.25–230)	0.696
Door-to-physician time (minutes)	5.81 ± 2.93 4.5 (4–7.5)	5.29 ± 2.98 5 (4–7)	0.465
Door-to-CT time (minutes)	21.39 ± 19.67 14.5 (12–21.25)	22.38 ± 18.96 16 (11.25–20.75)	0.624
Door-in-door-out time (minutes)	50.42 ± 18.55 45 (40–53.5)	49.41 ± 18.34 45 (39–51.75)	0.639

For numerical: mean ± SD; median (Q1–Q3).

**Table 4:** Laboratory results at admission (*n* = 132).

Variables	Sample		*p* Value
	Long COVID (*n* = 26)	No Long COVID (*n* = 106)	
Laboratory results			
Hemoglobin, mg/dL	13.52 ± 1.74 13.55 (12.425–14.8)	13.26 ± 1.51 13.45 (12.2–14.48)	0.554
Platelets count, ×109 μL	218.27 ± 69.39 204.5 (162.5–246)	221.58 ± 73.59 223.5 (175.3–251.8)	0.593
Blood sugar level, mg/dL	126.15 ± 30.02 118.5 (106.75–137.75)	134.38 ± 47.44 119.5 (100.5–153)	0.823
INR	0.86 ± 0.06 0.9 (0.8–0.9)	0.89 ± 0.08 0.9 (0.8–0.9)	0.093
Partial thromboplastin time, seconds	33.39 ± 2.16 33 (31.5–35)	33.01 ± 2.83 33.5 (31–35)	0.878

INR, international normalized ratio. Values were expressed as mean ± standard deviation (SD); Mann–Whitney U Test for continuous variable without Gaussian distribution—data represented by median (interquartile range).

**Table 5:** Treatment and outcomes for all patients (*n* = 132).

Association Variables		Sample		*p* Value
		Long COVID (*n* = 26)	No Long COVID (*n* = 106)	
**Treatment**				
Treatment	No treatment	16 (61.5%)	75 (70.8%)	0.205
	Intravenous thrombolysis	2 (7.7%)	14 (13.2%)	
	Mechanical thrombectomy	8 (30.8%)	17 (16.0%)	
**Outcomes**				
Discharge		24 (92.3%)	95 (90.1%)	1.000
Hospitalization days		8.27 ± 2.24 8.5 (7–9)	8.38 ± 2.73 8 (7–9)	0.956
Hemorrhagic transformation		7 (26.9%)	15 (14.2%)	0.143

For numerical: mean ± SD; median (Q1–Q3), and for nominal: *n* (%).

## Data Availability

The datasets are not publicly available, but de-identified data may be provided upon request from Florina Buleu.
